# Circulating Soluble Suppression of Tumorigenicity 2 Predicts Recurrence After Radiofrequency Ablation of Persistent Atrial Fibrillation

**DOI:** 10.3389/fcvm.2021.653312

**Published:** 2021-06-17

**Authors:** Ruopeng Tan, Haixu Yu, Xu Han, Yang Liu, Xiaolei Yang, Yun-Long Xia, Xiaomeng Yin

**Affiliations:** ^1^Department of Cardiology, The First Affiliated Hospital of Dalian Medical University, Dalian, China; ^2^Institute of Cardiovascular Diseases, The First Affiliated Hospital of Dalian Medical University, Dalian, China; ^3^Department of Cardiology, Peking University Third Hospital, Beijing, China

**Keywords:** atrial fibrillation, atrial fibrosis, sST2, biomarker, recurrence

## Abstract

**Objective:** A more extensively fibrotic left atrium contributes to atrial fibrillation (AF) occurrence, persistence, and recurrence. The soluble suppression of tumorigenicity 2 (sST2) has emerged as a ventricular fibrotic biomarker for patients with heart failure. The present study is to investigate associations between circulating sST2 and risk of recurrence after ablation in AF patients.

**Methods:** We measured the baseline plasma level of sST2 from patients with persistent AF (*n* = 117) and paroxysmal AF (*n* = 93) patients. Patients were followed up for 15 months after ablation. The relationship between circulating sST2 and recurrence was assessed by multivariable Cox regression. The cutoff value of sST2 was determined by receiver operating characteristic curve. The relationship between baseline sST2 level and left atrial volume index (LAVI) was assessed by multivariate linear regression analysis. Serial sST2 measurements were also conducted after 24 h, 6 months, and 15 months of ablation. ST2 localization was examined in left atrial appendages of persistent AF patients by immunohistochemistry and Western blot.

**Results:** Baseline sST2 positively associated with LAVI in the persistent AF group, and elevated sST2 (≥39.25 ng/ml) independently increased the risk of recurrence after ablation (area under the curve = 0.748), with hazard ratio of 1.038 (95% confidence interval 1.017–1.060, *P* < 0.001) when adjusted for co-variables. In contrast, elevated sST2 cannot predict recurrence in paroxysmal AF.

**Conclusions:** In persistent AF patients, increased sST2 serves as a marker of recurrence after radiofrequency ablation. Patients with sST2 ≥ 39.25 ng/ml are more likely to develop recurrence within a year.

## Introduction

Atrial fibrillation (AF) is the most common arrhythmia and contributes significantly to mortality and morbidity (1, 2). Large clinical trials have shown that there are no currently available anti-arrhythmic medications in the treatment for AF (3, 4). Despite the success of using catheter ablation in the control of AF, recurrence rates still remain high, ranging from 30 to 60% across reports (5, 6). A more extensively fibrotic left atrium contributes to AF occurrence, persistence, and recurrence (7, 8). However, a valuable circulating marker, which can predict atrial fibrosis or recurrence following ablation is very limited.

Suppression of tumorigenicity 2 (ST2) belongs to the Toll-like/IL-1 receptor superfamily. ST2 has two main isoforms, the soluble ST2 (sST2) and the membrane-bound ST2 (ST2L), due to differential promoter binding (9, 10). Binding of ST2L and IL-33 promotes NF-κB pathway activation (9). sST2 lacks the transmembrane and cytoplasmic domains in comparison with ST2L, and functions as a decoy receptor to prevent the binding between ST2L and IL-33 (11). Membrane receptor ST2L is mainly expressed on hematopoietic cells and involved in inflammatory responses (9). sST2 is induced in fibroblasts or cardiomyocytes subjected to stimulations (12). sST2 has emerged as a ventricular fibrotic biomarker for patients with heart failure or myocardial infarction, and the initial sST2 level was independently associated with the incidence of mortality (13, 14).

Blood sST2 concentration in AF patients was higher than that of healthy controls, and sST2 was likely a biomarker, which can predict AF patients' risk of emergency admission (15). Circulating sST2 can predict the risk of occurrence of the new-onset AF with coronary artery disease (16). Okar et al. also reported that sST2 was an independent parameter for predicting AF recurrence in patients with non-valvular paroxysmal AF who have undergone cryoballoon catheter ablation (17). Moreover, Walek et al. reported that sST2 was a predictor of successful electrical cardioversion and long-term maintenance of sinus rhythm (SR) in persistent AF patients (18). Wang et al. reported that sST2 levels were higher in paroxysmal AF patients with left atrial low-voltage zone (LVZ) > 20% compared with those with a smaller LVZ, and elevated sST2 levels served as a novel predictor of paroxysmal AF recurrence rate in patients who had undergone ablation (19). Persistent AF patients normally have a more severe fibrotic atrium than paroxysmal AF patients (20), which promote us to investigate the diagnostic value of sST2 in the setting of persistent AF.

In the present study, we measured the baseline circulating level of sST2 in patients with paroxysmal AF or persistent AF, and examined whether sST2 could be an important indicator of AF recurrence following ablation. Moreover, additional experiments were also performed to explore the potential cellular sources of sST2 in AF patients.

## Methods

### Study Population

The current study was reviewed and approved by the Institutional Review Board of the First Affiliated Hospital of Dalian Medical University, and conducted in accordance with the Declaration of Helsinki. All the participants signed an informed consent. Initially, a total of 356 consecutive patients with drug-refractory AF who underwent their primary radiofrequency catheter ablation procedure at our hospital were recruited to the study between January 2015 and December 2018. AF patients were categorized as paroxysmal when episodes self-terminated within 7 days or as persistent when episodes lasted over 7 days or required electrical cardioversion. Patients were excluded from the study if (1) they had received a previous endovascular, surgical AF ablation or a maze surgery, (2) they had untreated hyperthyroidism, (3) they had heart failure [left ventricular ejection fraction (LVEF) < 50% or B-type natriuretic peptide (BNP) > 400 ng/ml], coronary heart disease, stroke, pulmonary diseases, and hepatic or renal dysfunction, (4) they were in chronic inflammatory status or had acute infection, and (5) patients needed cardioversion post ablation. Finally, a total of 210 patients were enrolled for the follow-up study.

### Radiofrequency Catheter Ablation

Oral anticoagulation therapy was stopped at least 1 day before ablation and bridged with low molecular weight heparin. Patients were heparinized to activate clotting time > 250 s, after double transseptal puncture. Circumferential pulmonary vein isolation (CPVI) using irrigated radiofrequency ablation is the initial ablation approach used in all AF patients who underwent ablation. For persistent AF patients, additional substrate modification, including left atrial linear ablation and isthmus ablation, were performed followed by CPVI. The endpoint of CPVI was defined as the absence of any pulmonary vein spike potential in the spiral-mapping catheter inside the lateral PVs. Ablation of complex fractionated atrial electrograms (CFAE) guided by an LA CFAE map was performed if SR could not be achieved after CPVI. If frequent atrial premature beats or atrial tachycardia occurred, superior vena cava isolation was performed. A cavotricuspid isthmus ablation was performed if familiar atrial flutter occurred (21).

### Assessment of Soluble Suppression of Tumorigenicity

Fresh blood samples (2 ml) were obtained by venipuncure from AF patients prior to ablation (baseline), 24 h, 6 months, and 15 months after ablation. Levels of sST2 were measured by using human sST2 enzyme-linked immunosorbant assay (ELISA) kit (Presage ST2 Assay, Critical Diagnostics, USA).

### Follow-Up

After the ablation, all patients were followed up for 15 months (the first 3 months is blanking period). In total, of 210 people initially recruited, 92.8% of the participants had their data measured at the end of this study. One patient was lost to follow-up at 8 months, and further 14 patients (6.66%) were lost to follow-up between 12 and 15 months. Amiodarone or propafenone were administered after ablation if not contraindicated to prevent the early recurrence of AF. Anticoagulation treatment was prescribed for at least 3 months and thereafter according to the CHA_2_DS_2_-VASC score. Patients were evaluated by 24-h Holter monitor if symptoms of arrhythmia occurred. Routine medical examination, including the 24-h Holter monitoring, was performed for patients without any symptoms at 3, 6, 9, 12, and 15 months post-ablation. Recurrence was defined as AF, atrial tachycardia, or atrial flutter ≥ 30 s in duration after a 3-month blanking period according to the 2012 Heart Rhythm Society consensus document.

### Immunohistochemistry

Atrial appendages from persistent AF patients with valvular disease were collected during valvular replacement surgery and then fixed in 4% paraformaldehyde (*n* = 6). In the control group, heart samples were taken from body donors who died in an accident and were anatomically confirmed to have no cardiovascular diseases (*n* = 6). After embedding in paraffin, samples were cut into 4-μm sections for HE staining, Masson staining, and IHC.

IHC was used to determine the contents of ST2 or α-SMA in the atrial sections. Briefly, antigen retrieval was conducted by immerging in the citrate-EDTA buffer and then in microwave oven for 5 min at high power. Non-specific staining was blocked by using 3% H_2_O_2_ and then followed by 10% goat serum. After blocking, 50 μl of diluted primary antibodies, sST2 (1/100 dilution, 11920-1-AP, Proteintech, Wuhan, China) or α-SMA (1/100 dilution, 14395-1-AP, Proteintech, Wuhan, China) were applied onto each section for 1 h. After incubation with biotin-conjugated-goat anti-rabbit secondary antibody (1/1,000 dilution, B-2770, Thermo Fisher, USA) sections were incubated with Avidin and NeutrAvidin™ Biotin-Binding complex (A2666, Thermo Fisher, USA) for 30 min. Finally, sections were visualized by DAB staining (A34002, Thermo Fisher, USA) for two (α-SMA) or 3 min (sST2).

### Western Blotting

Total protein from atrial appendages was isolated with radioimmunoprecipitation assay (RIPA) buffer (Beytime, China). Equal amounts (30 μg) of protein from samples were loaded on SDS-Page and run at constant voltage about 80 V for 1 h through the resolving gel and at 120 V for 2.5 h through the stacking gel. After transfer, the membranes were blocked with 5% skim milk for 1 h and then incubated in diluted primary antibody ST2 (1/1,000 dilution, 11920-1-AP, Proteintech, Wuhan, China) at 4°C overnight. After the primary antibody incubation, membranes were washed three times (each for 10 min) in TBST and then incubated in a goat-anti rabbit secondary antibody conjugated with horseradish peroxidase (1/10,000, A16096, Thermo Fisher, USA) for 2 h at room temperature. Membranes were then washed three times before exposure using Immun-star HRP chemiluminescent substrate kit (1705040, Bio-Rad, USA). After exposure, the antibodies loaded on the membranes were stripped in stripping solution (0.0625 M Tris-Cl pH 6.8; 2% SDS, 0.7% β-mercaptoethanol) for 30 min at 60°C. Then membranes were re-probed with α-tubulin (1/5,000, ab4074, Abcam, USA) at 4°C overnight to verify loading consistency.

### Statistical Analysis

Continuous data were tested for normality and expressed as mean ± standard deviation (SD). Qualitative variables were expressed as percentages (%), and Fisher's exact test and χ^2^ test were used for comparison between groups. Hazard ratios (HR) with 95% confidence intervals (CI) were presented. Associations between circulating sST2 and left atrial volume index (LAVI) were assessed by multivariable linear regression models after adjusting for co-variables. Univariate and multivariable Cox proportional hazard regression were used to identify significant predictors of the primary outcomes. The associations between sST2 level and AF recurrence were analyzed by multivariable Cox regression analysis. Receiver operating characteristic curve (ROC) curves were developed using a probability-weighted Cox model. Recurrence rate in AF patients following ablation was visualized using Kaplan–Meier survival curves and Log-rank tests. Serial measurements of sST2 were analyzed using repeated measurements analyses of variance. All values were two-tailed, and differences were considered statistically significant at *P* < 0.05. Gpower software version 3.1 (Gpower Kiel, Germany) was used to perform power analysis; the total estimated sample size in our study was 172. Power, alpha, and effect size were set at 90%, 0.05, and 0.5, respectively. The statistical analysis was performed with SPSS software version 13.0 (SPSS Inc., Chicago, IL, USA).

## Results

### Demographic and Clinical Characteristics

**Figure 1:**
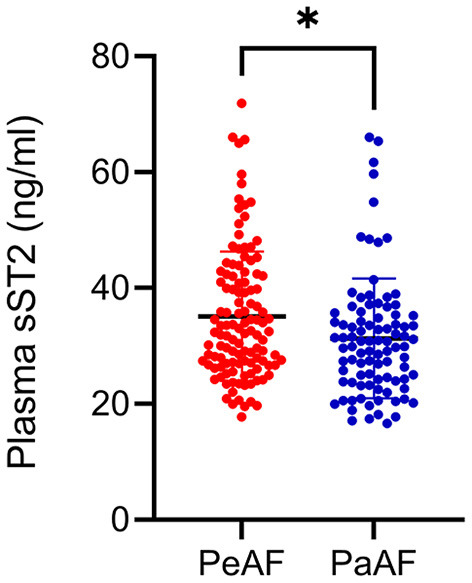
Enhanced plasma soluble suppression of tumorigenicity 2 (sST2) was found in persistent atrial fibrillation (AF) patients. Plasma levels of sST2 were measured by enzyme-linked immunosorbant assay (ELISA) kit and presented by dot plots. PaAF, paroxysmal AF; PeAF, persistent AF. *P < 0.01 vs. PeAF.

Table 1 summarizes the baseline characteristics of all the participants. A total of 210 AF patients (117 persistent and 93 paroxysmal AF patients) were enrolled in this study. AF recurrence (20.5%) was observed between 3 and 15 months after ablation. The occurrence of AF was predominant in male. The most prevalent comorbidity was hypertension (41.9%), followed by hyperlipidemia (24.8%) and T2DM (12.4%). Significant difference in the use of beta blockers and the plasma level of sST2 was observed. Table 2 summarizes the baseline characteristics of the persistent AF patients. During a total of 15-month follow-up period, 80.3% (94/117) persistent AF patients were successfully converted to SR. We further confirmed that circulating sST2 from persistent AF patients was significantly increased compared with that from paroxysmal AF patients at baseline level (Figure 1).

**Table 1:** Clinical characteristics in all atrial fibrillation (AF) patients.

**Variables**	**Recurrence** **(*n* = 43)**	**No recurrence** **(*n* = 167)**	***P*-value**
Age (years)	57.74 ± 11.45	58.49 ± 9.95	0.674
Male (*n*, %)	26 (60.5%)	117 (70.1%)	0.271
History of AF (months)	4.84 ± 4.17	4.76 ± 4.86	0.837
Persistent AF (*n*, %)	33 (57.9%)	84 (54.9%)	0.756
Smoking (*n*, %)	15 (34.9%)	47 (28.1%)	0.454
**Medical history**			
Hyperlipidemia (*n*, %)	14 (32.6%)	38 (22.8%)	0.234
Hypertension (*n*, %)	20 (46.5%)	68 (40.7%)	0.494
T2DM (*n*, %)	2 (4.7%)	24 (14.4%)	0.118
**Medication**			
VKA (*n*, %)	14 (32.6%)	35 (21.0%)	0.156
Amiodarone (*n*, %)	22 (51.2%)	92 (55.1%)	0.732
Beta blockers (*n*, %)	10 (23.3%)	18 (10.8%)	0.043
ACEI (*n*, %)	3 (7.0%)	6 (3.6%)	0.394
**Lab tests of plasma**			
sST2 (ng/ml)	38.65 ± 17.78	32.44 ± 10.34	0.033
Cre (μmol/L)	79.00 ± 47.71	71.98 ± 14.27	0.346
UA (μmol/L)	366.43 ± 122.69	360.71 ± 79.97	0.756
FBG (g/L)	5.82 ± 1.42	5.91 ± 1.17	0.673
TC (mmol/L)	4.78 ± 1.04	4.77 ± 1.08	0.982
HDL (mmol/L)	1.25 ± 0.26	1.23 ± 0.25	0.603
HCY (μmol/L)	12.83 ± 5.79	14.59 ± 7.92	0.177
TSH (mU/L)	2.37 ± 1.84	2.37 ± 1.78	0.988
**Echocardiography**			
LVEF (%)	63.38 ± 6.31	61.60 ± 7.11	0.137
LAVI (ml/m^2^)	30.74 ± 7.54	27.29 ± 7.76	0.009
**Ablation time (min)**	88.56 ± 20.74	94.59 ± 24.23	0.136

*T2DM, type 2 diabetes mellitus; VKA, vitamin K antagonist; ACEI, angiotensin-converting enzyme inhibitors; sST2, soluble suppression of tumorigenicity 2; Cre, creatinine; UA, uric acid; FBG, fibrinogen; TC, total cholesterol; HDL, high-density lipoprotein; HCY, homocysteine; TSH, thyroid stimulating hormone; LVEF, left ventricular ejection fraction; LAVI, left atrial volume index*.

**Table 2:** Clinical characteristics in persistent AF patients.

**Variables**	**Recurrence** **(*n* = 23)**	**No recurrence** **(*n* = 94)**	***P*-value**
Age (years)	57.70 ± 12.89	59.03 ± 9.60	0.578
Male (*n*, %)	16 (69.6%)	73 (77.7%)	0.423
History of AF (years)	4.79 ± 4.47	4.60 ± 5.18	0.871
Smoking	7 (30.4%)	26 (27.7%)	0.800
**Medical history**			
Hyperlipidemia (*n*, %)	6 (26.1%)	17 (18.1%)	0.390
Hypertension (*n*, %)	10 (43.5%)	39 (41.5%)	0.862
T2DM (*n*, %)	0 (0%)	9 (9.6%)	0.202
**Medication**			
VKA (*n*, %)	13 (56.5%)	28 (29.8%)	0.027
Amiodarone (*n*, %)	18 (78.3%)	74 (78.7%)	0.961
Beta blockers (*n*, %)	6 (26.1%)	11 (11.7%)	0.100
ACEI (*n*, %)	3 (13.0%)	5 (5.3%)	0.189
**Lab tests of plasma**			
sST2 (ng/ml)	46.38 ± 20.58	33.46 ± 10.32	0.007
Cre (μmol/L)	87.05 ± 63.25	72.56 ± 13.26	0.286
UA (μmol/L)	403.35 ± 124.28	380.56 ± 77.44	0.408
FBG (g/L)	5.42 ± 0.72	5.77 ± 0.96	0.112
TC (mmol/L)	4.73 ± 1.24	4.85 ± 1.24	0.685
HDL (mmol/L)	1.22 ± 0.24	1.23 ± 0.25	0.837
**Echocardiography**			
LVEF (%)	61.18 ± 5.50	59.45 ± 7.30	0.359
LAVI (ml/m^2^)	33.82 ± 6.62	28.52 ± 8.34	0.005
**Ablation time (min)**	101.30 ± 19.37	108.61 ± 22.81	0.126

*T2DM, type 2 diabetes mellitus; VKA, vitamin K antagonist; ACEI, angiotensin-converting enzyme inhibitors; sST2, soluble suppression of tumorigenicity 2; Cre, creatinine; UA, uric acid; FBG, fibrinogen; TC, total cholesterol; HDL, high-density lipoprotein; HCY, homocysteine; TSH, thyroid stimulating hormone; LVEF, left ventricular ejection fraction; LAVI, left atrial volume index*.

### Baseline Soluble Suppression of Tumorigenicity 2 Correlated to Left Atrial Volume Index and Atrial Fibrillation Recurrence After Ablation in Persistent Atrial Fibrillation Patients

**Figure 2:**
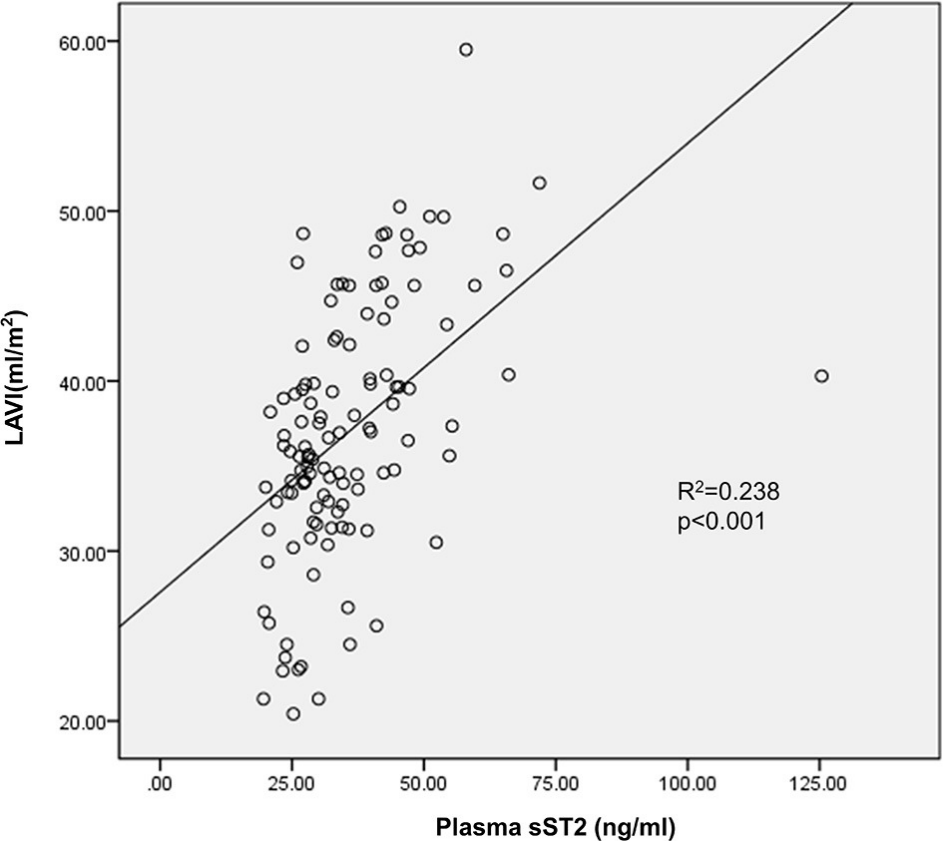
Baseline level of circulating sST2 linked to left atrial volume index (LAVI) in persistent AF patients. Multivariate linear regression analyzed the correlation between baseline sST2 and LAVI.

Both univariate and multivariate linear regression models were used to evaluate relationships between baseline sST2 and LAVI in persistent AF patients (Figure 2, Tables 3, 4). Only baseline level of sST2 reached a *P*-value of < 0.05 during the univariate linear regression analysis. For the multivariate linear regression model, all covariates listed in Table 1 were adjusted. Baseline sST2 was strongly associated with LAVI during the univariate linear regression analysis (Table 4). The relationships between sST2 and LAVI were also evaluated in paroxysmal AF patients, with no significant differences (Table 5).

**Table 3:** Univariate linear regression analyzed the correlations between baseline sST2 and LAVI in persistent AF patients.

**Variables**	**Unstandardized coefficients**		**Standardized coefficients**	***t***	***P***	**95% CI for B**	
	**B**	**SE**	**B**			**Lower**	**Upper**
Age (years)	0.058	0.068	0.079	0.848	0.398	−0.077	0.192
Male (*n*, %)	−0.824	1.631	−0.047	−0.505	0.614	−4.056	2.407
History of AF (months)	−0.124	0.139	−0.083	−0.891	0.375	−0.398	0.151
Smoking (*n*, %)	−1.575	1.541	−0.095	−1.022	0.309	−4.628	1.479
Hyperlipidemia (*n*, %)	−0.224	1.753	−0.012	−0.128	0.899	−3.696	3.249
Hypertension (*n*, %)	−1.149	1.408	−0.076	−0.816	0.416	−3.938	1.641
T2DM (*n*, %)	0.213	2.615	0.008	0.82	0.935	−4.966	5.393
VKA (*n*, %)	2.618	1.440	0.167	1.818	0.072	−0.234	5.47
Amiodarone (*n*, %)	0.289	1.700	0.016	0.170	0.865	−3.077	3.656
Beta blockers (*n*, %)	3.437	1.951	0.162	1.761	0.081	−0.428	7.301
ACEI (*n*, %)	2.503	2.751	0.085	0.91	0.365	−2.946	7.952
sST2 (ng/ml)	0.264	0.044	0.488	5.995	<0.001	0.177	0.351
Cre (μmol/L)	0.004	0.023	0.017	0.186	0.853	−0.041	0.050
UA (μmol/L)	0.003	0.008	0.041	0.435	0.664	−0.012	0.019
FBG (g/L)	0.551	0.746	0.070	0.738	0.462	−0.928	2.029
TC (mmol/L)	0.133	0.567	0.022	0.235	0.815	−0.990	1.256
HDL (mmol/L)	−1.087	2.783	−0.037	−0.390	0.697	−6.601	4.428
HCY (μmol/L)	−0.090	0.093	−0.091	−0.968	0.335	−0.275	0.994
TSH (mU/L)	0.455	0.396	0.108	1.149	0.253	−0.330	1.241
LVEF (%)	−0.093	0.104	−0.088	−0.892	0.374	−0.299	0.113

*T2DM, type 2 diabetes mellitus; VKA, vitamin K antagonist; ACEI, angiotensin-converting enzyme inhibitors; sST2, soluble suppression of tumorigenicity 2; Cre, creatinine; UA, uric acid; FBG, fibrinogen; TC, total cholesterol; HDL, high-density lipoprotein; HCY, homocysteine; TSH, thyroid stimulating hormone; LVEF, left ventricular ejection fraction; LAVI, left atrial volume index*.

**Table 4:** Multivariate linear regressions for the correlations between baseline sST2 and LAVI in persistent AF patients.

**Variables**	**Unstandardized coefficients**		**Standardized coefficients**	***t***	***P***	**95% CI for B**	
	**B**	**SE**	**B**			**Lower**	**Upper**
sST2 (ng/ml)	0.257	0.055	0.499	0.711	<0.001	0.149	0.366

*sST2, soluble suppression of tumorigenicity 2*.

**Table 5:** Univariate linear regression analyzed the correlations between baseline sST2 and LAVI in paroxysmal AF patients.

**Variables**	**Unstandardized coefficients**		**Standardized coefficients**	***t***	***P***	**95% CI for B**	
	**B**	**SE**	**B**			**Lower**	**Upper**
Age (years)	0.128	0.068	0.195	1.895	0.061	−0.006	0.262
Male (*n*, %)	1.218	1.418	0.09	0.859	0.393	−1.599	4.034
History of AF (months)	0.136	0.163	0.087	0.834	0.407	−0.188	0.46
Smoking (*n*, %)	−1.742	1.505	−0.12	−1.157	0.25	−4.732	1.248
Hyperlipidemia (*n*, %)	2.255	1.498	0.156	1.506	0.136	−0.72	5.23
Hypertension (*n*, %)	1.027	1.419	0.076	0.723	0.471	−1.793	3.846
T2DM (*n*, %)	1.182	2.092	0.059	0.565	0.574	−2.974	5.337
VKA (*n*, %)	−1.972	2.497	−0.08	−0.790	0.432	−6.931	2.987
Amiodarone (*n*, %)	1.184	1.648	0.075	0.718	0.474	−2.09	4.458
Beta blockers (*n*, %)	−1.59	2.169	−0.077	−0.733	0.465	−5.898	2.718
ACEI (*n*, %)	1.825	6.808	0.028	0.268	0.789	−11.698	15.347
sST2 (ng/ml)	0.103	0.072	0.149	1.439	0.153	−0.039	0.246
Cre (μmol/L)	−0.032	0.046	−0.073	−0.696	0.488	−0.123	0.059
UA (μmol/L)	−0.005	0.009	−0.052	−0.501	0.617	−0.023	0.014
FBG (g/L)	−0.198	0.478	−0.043	−0.413	0.681	−1.15	0.754
TC (mmol/L)	0.512	0.86	0.062	0.596	0.553	−1.196	2.221
HDL (mmol/L)	1.846	2.775	0.070	0.665	0.508	−3.666	7.358
HCY (μmol/L)	0.011	0.107	0.011	0.102	0.919	−0.201	0.223
TSH (mU/L)	0.858	0.445	0.199	1.931	0.057	−0.025	1.742
LVEF (%)	−0.249	0.116	−0.222	−2.150	0.034	−0.479	-0.019

*T2DM, type 2 diabetes mellitus; VKA, vitamin K antagonist; ACEI, angiotensin-converting enzyme inhibitors; sST2, soluble suppression of tumorigenicity 2; Cre, creatinine; UA, uric acid; FBG, fibrinogen; TC, total cholesterol; HDL, high-density lipoprotein; HCY, homocysteine; TSH, thyroid stimulating hormone; LVEF, left ventricular ejection fraction; LAVI, left atrial volume index*.

The associations between baseline sST2 level and all AF recurrence were analyzed by multivariable Cox regression analysis based on adjusted for nothing (Model 1); sex and age (Model 2); and sex, age, hypertension, type 2 diabetes mellitus (T2DM), uric acid (UA), total cholesterol (TC), and beta blockers (Model 3) (Table 6). The associations between baseline sST2 and paroxysmal AF or persistent AF were based on adjusted for sex, age, hypertension, T2DM, UA, TC, and beta blockers (Model 4 and Model 5; Table 6). After adjusting for potential confounders, we found that sST2 was an independent predictor of recurrence for all AF patients (HR = 1.026, 95% CI 1.007–1.046, *P* < 0.001), especially in the persistent AF group (HR = 1.038, 95% CI 1.017–1.060, *P* < 0.001) (Table 2), but not in paroxysmal AF patients.

**Table 6:** Associations between sST2 and AF recurrence by Cox Regression.

	**HR (95% CI)**	***P*-value**
Model 1	1.030 (1.013–1.047)	<0.001
Model 2	1.033 (1.016–1.050)	<0.001
Model 3	1.026 (1.007–1.046)	0.008
Model 4	0.978 (0.929–1.031)	0.41
Model 5	1.038 (1.017–1.060)	<0.001

*Model 1 adjusted for nothing*.

*Model 2 adjusted for sex and age*.

*Model 3 adjusted for sex, age, hypertension, T2DM, UA, TC, beta blockers, and LAVI*.

*Model 4 adjusted for sex, age, hypertension, T2DM, UA, TC, beta blockers, and LAVI in PaAF*.

*Model 5 adjusted for sex, age, hypertension, T2DM, UA, TC, beta blockers, and LAVI in PeAF*.

*HTN, hypertension; T2DM, Type 2 diabetes mellitus; UA, uric acid; TC, total cholesterol; PaAF, paroxysmal atrial fibrillation; PeAF, persistent atrial fibrillation; LAVI, left atrial volume index; HR, hazard ratios; CI, confidence intervals*.

**Figure 3:**
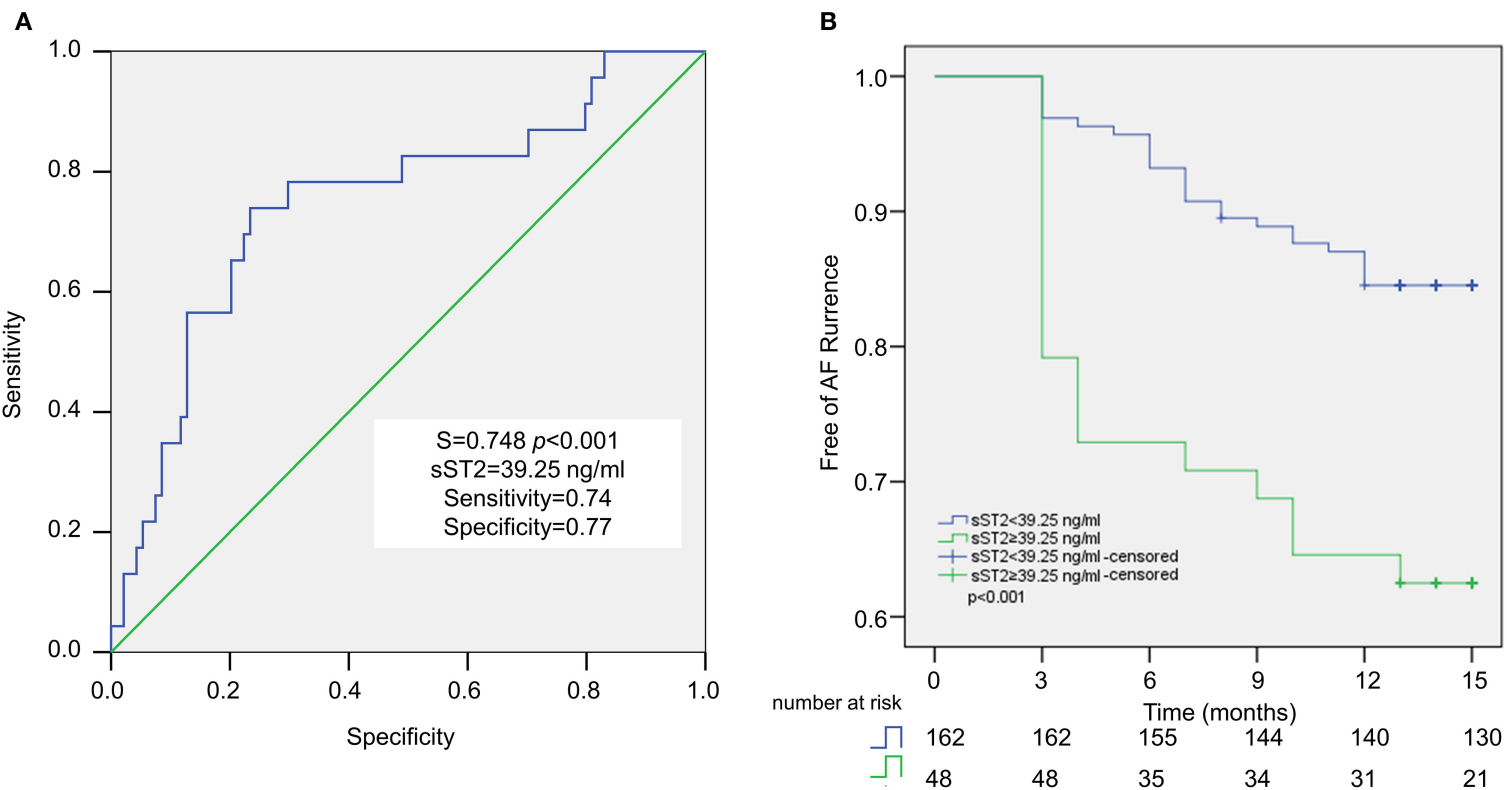
Baseline sST2 for the prediction of AF recurrence after ablation in persistent AF patients. Receiver operating characteristic (ROC) curve analysis revealed the cutoff value for sST2 is 39.25 ng/ml, with sensitivity at 74%, and specificity at 77% **(A)**. Recurrence rate after ablation is examined by Kaplan–Meier analysis **(B)**.

ROC analysis demonstrated that plasma sST2 ≥ 39.25 ng/ml was predictive of recurrence after a single ablation, with 74% sensitivity and 77% specificity. The area under the ROC curve was 0.748 (Figure 3A). Kaplan–Meier analysis showed a significantly higher rate of recurrent AF in sST2 high (17 of 39, 43.6%) vs. sST2 low group (6 of 78, 7.7%) (Figure 3B).

### Serial Soluble Suppression of Tumorigenicity 2 Measurements at Different Time Points

**Figure 4:**
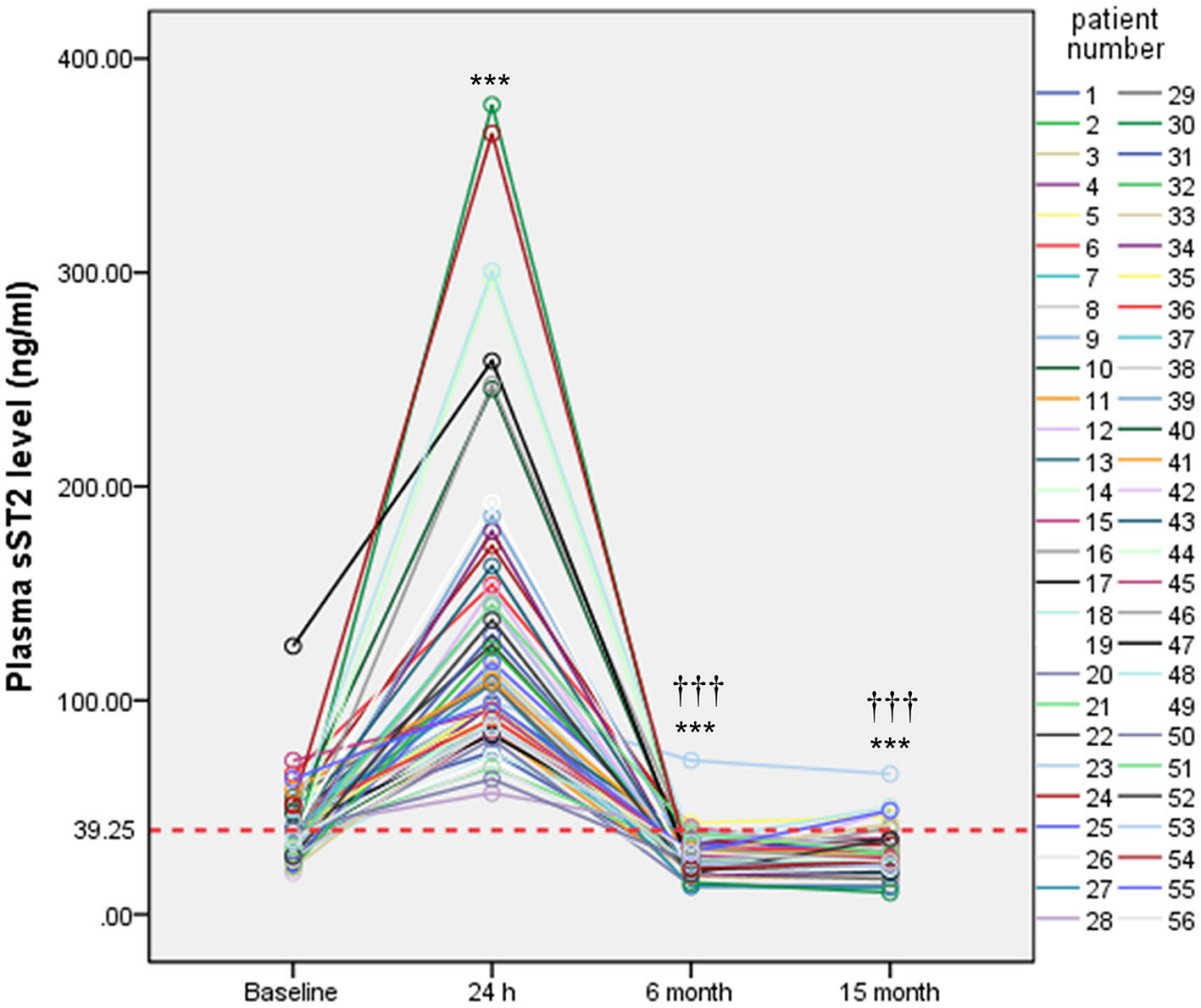
Serial measurements of plasma sST2 in persistent AF patients. Plasma levels of sST2 were measured at baseline, 24 h, 6 months, and 15 months after ablation and presented by different color lines. Red lines indicate threshold value for diagnosis. Statistical analysis was performed by repeated measurements analyses of variance. ***P < 0.001 vs. baseline;^†††^*P* < 0.001 vs. 24 h.

sST2 levels in persistent AF patients were measured at four different time points. After 24 h of ablation, sST2 was dramatically increased. After 6 months of ablation, sST2 level dropped below the baseline level. There was no change between 6 and 15 months of post ablation in sST2 levels (Figure 4).

### Accumulation of Soluble Suppression of Tumorigenicity 2 Was Observed in Atria of Persistent Atrial Fibrillation Patients

**Figure 5:**
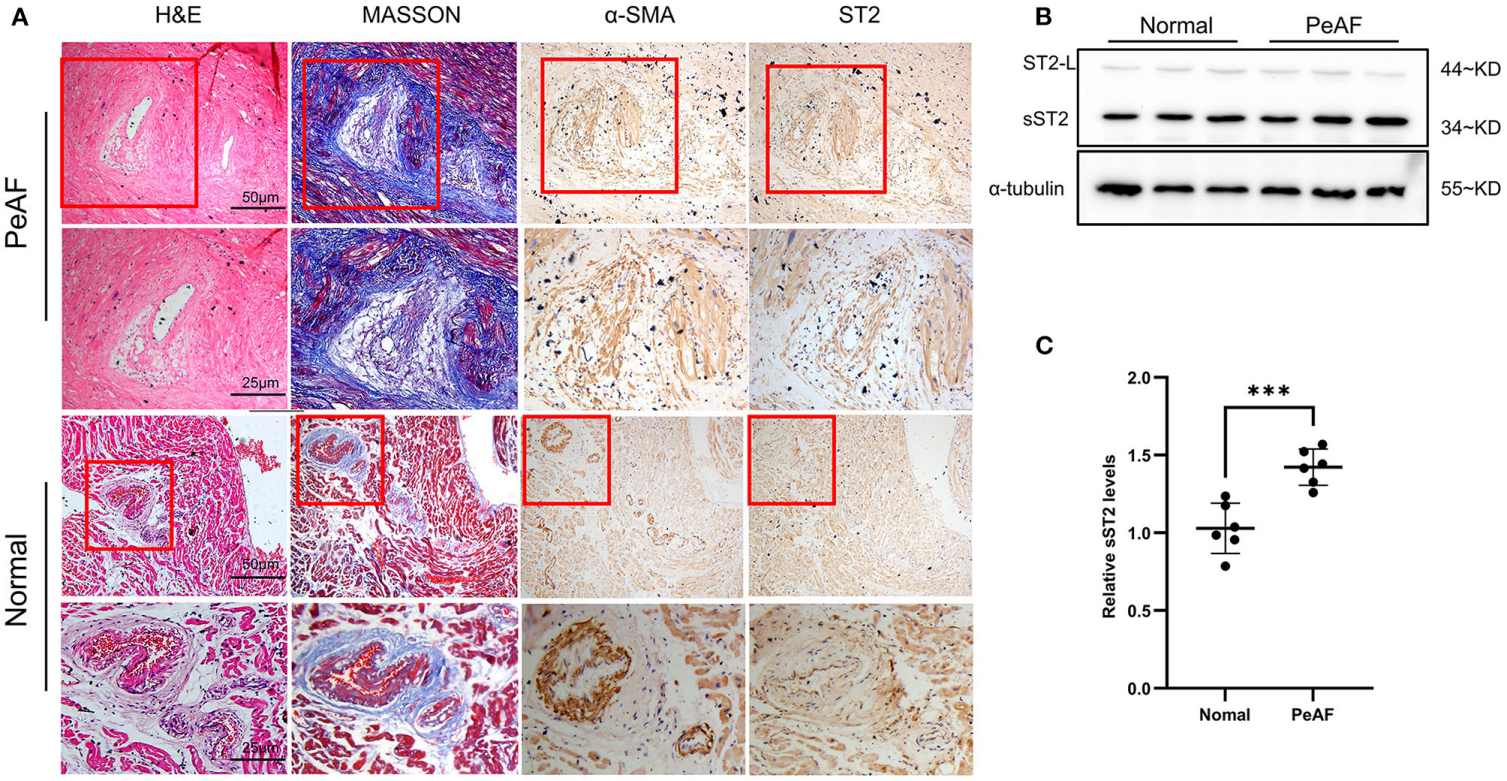
Enhanced sST2 production in atria from persistent AF patients. Representative images of Masson, HE, and immunohistochemistry (IHC) staining (α-SMA and sST2) in atria from persistent AF patients and sinus rhythm (SR) controls at low and high magnifications **(A)**. IHC staining showing that sST2 and fibrotic tissues are restricted in the similar areas (indicated by red squares) **(A)**. The content of ST2 in atria was measured by Western blotting **(B)**. Quantitation is shown in **(C)**. PeAF, persistent AF. ***P < 0.001 vs. Baseline.

Atria from persistent AF patients displayed high level of fibrotic areas, as indicated by Masson, HE, and IHC staining (α-SMA) (Figure 5A). Interestingly, IHC results revealed that ST2 was largely found in the fibrotic area, that co-localized with myofibroblasts (Figure 5A), indicating that cardiac ST2 was mainly derived from myofibroblasts. Western blot confirmed that cardiac ST2 was mainly in the form of sST2, not ST2L, according to the molecular weight (Figures 5B,C).

## Discussion

The major findings in the current study were (1) baseline sST2 from persistent AF patients was elevated compared with paroxysmal AF patients; (2) baseline sST2 positively correlated with LAVI and was able to predict recurrence after ablation in persistent AF patients; and (3) atrial fibroblasts were likely a cellular source of circulating sST2. Our results provide a useful circulating marker for assessing response to catheter ablation and advance our understanding of the mechanisms of LA fibrosis.

Mechanistically, clinical and experimental studies have shown that atrial fibrosis is a prominent feature in AF, and is considered as the main mechanism of AF occurrence, persistence, and recurrence (7, 8). Therefore, circulating biomarkers, which reflect the degree of fibrosis could be considered as biomarkers of relapse after ablation. sST2 served as a ventricular fibrotic biomarker for patients with heart failure or myocardial infarction, and the initial sST2 level was independently associated with the incidence of mortality (13, 14). In the present study, we observed that baseline sST2 was elevated in persistent AF patients, and related to atrial structural remodeling, which was assessed by LAVI. Additionally, sST2 served as a strong predictor of recurrence for persistent AF patients, but not in paroxysmal AF. Liu et al. found that sST2 had poor correlation with the preexisted abnormalities during endocardial mapping, but well with the dynamic abnormalities during endocardial mapping (22). Their findings suggest that baseline sST2 level might have a role in distinguishing whether recurrence is originated from pulmonary vein or atrial fibrosis, and help in refining the future approach to AF ablation. In our study, whether the recurrent persistent AF patients who with high level of sST2 have additional abnormalities than recurrent persistent AF patients without, still needs further investigations.

Both ST2 forms are expressed in cardiac tissues (12). It was previously thought that the increase in circulating sST2 in AF patients is caused by higher heart rate and atrial pressure (23). In the present study, we confirmed that sST2 in the atria of persistent AF patients was much higher than the form of ST2L. Furthermore, we found that cardiac sST2 was mainly located in atrial myofibroblasts, suggesting that the increment of baseline sST2 in persistent AF may be a result of myofibroblast activation rather than higher heart rate and atrial pressure. Persistent AF patients are normally associated with more severe atrial fibrosis than paroxysmal AF patients (24), indicating that the number and activity of myofibroblasts are enhanced in the atria from persistent AF patients. This finding somehow explains why sST2 is preferential in predicting persistent AF rather than paroxysmal AF.

Furthermore, serial measurements of sST2 were conducted at three different time points in persistent AF patients. The value of sST2 at 24 h after ablation was dramatically increased compared with baseline value. Such a sudden increase was largely due to myocardial strain or damage during the ablation procedure. sST2 value dropped below baseline after 6 and 15 months of ablation, indicating that ablation was useful in repressing the release of sST2 from the heart. One study reported that measuring baseline sST2 would be a simple method in identifying which AF patients are at high risk of heart failure (23). Further research is needed to determine sST2 at which time points could be used as a biomarker for predicting heart failure in the future.

ST2L and sST2 exert opposite functions driven by binding with IL-33 (25, 26). Both cardiomyocytes and cardiac fibroblasts expressed IL-33 and sST2, and were increased by biomechanical strain and Ang II stimulation (12). In the mouse study, pressure overload model enhanced IL-33 protein production in the fibroblasts of left ventricle (12). Deletion of ST2 in mouse with pressure overload enhanced hypertrophy and cardiac fibrosis (12). Addition of IL-33 prevented cardiac fibrosis, improved cardiac function and survival after ischemia–reperfusion in rats through induction of anti-apoptotic proteins in cardiomyocytes (12). These results indicate that IL-33/ST2L system is cardiac protective. As a decoy receptor, sST2 blocks the binding between IL-33 and ST2L, which brings detrimental effects to the heart (26, 27). Treatment of rat cardiac fibroblasts with IL-33 was found to repress the migratory activity of fibroblasts (12, 28). In our present study, we found that atrial myofibroblast-derived sST2 was increased in persistent AF patients, compared with SR controls, which raised the possibility that the ST2/IL-33 system might regulate fibroblast activity directly, although the mechanisms need to be further investigated.

It is well-known that AF is associated with both regional and systemic inflammatory responses (29), which is characterized by upregulation of inflammatory mediators and enhanced leukocyte activity, especially monocytes/macrophages (29–31). Human macrophages constitutively expressed both ST2L and sST2. However, shifting these macrophages toward an M2 phenotype by using IL-4 and IL-13 increased the expression of ST2L but not sST2 (32). Activation of ST2L/IL-33 signaling enhanced activation of mouse peritoneal macrophages (33). Whether elevated circulating sST2 in AF patients could affect monocyte/macrophage phenotype or activity still needs further investigation.

## Limitations

We have several limitations in the present study. *First*, our study was carried out in one center and only included a small number of patients. *Second*, the IHC and WB experiments were done on left atrial appendages taken from patients presenting with a different pathology (AF combined with valvular heart disease). *Third*, although echocardiography was used to evaluate atrial structural remodeling, there is no direct assessment of atrial fibrosis by using MRI.

## Conclusion

In persistent AF, circulating sST2 ≥ 39.25 ng/ml was predictive of recurrence after primary ablation. Furthermore, atrial myofibroblasts were likely a cellular source of circulating sST2, which might serve as an important biomarker for the degree atrial fibrosis. Inhibiting circulating sST2 might be useful as an adjuvant treatment to improve outcomes of catheter ablation for persistent AF.

## Data Availability Statement

The raw data supporting the conclusions of this article will be made available by the authors, without undue reservation.

## Ethics Statement

The studies involving human participants were reviewed and approved by the ethics committee of the First Affiliated Hospital of Dalian Medical University. The patients/participants provided their written informed consent to participate in this study.

## Author Contributions

XYi and Y-LX designed the research. RT, HY, XH, XYa, and YL carried out the experiments. RT, XH, XYa, and YL analyzed the results. XYi and Y-LX wrote the paper. RT, YL, and XYi revised the manuscript. All authors contributed to the article and approved the submitted version.

## Conflict of Interest

The authors declare that the research was conducted in the absence of any commercial or financial relationships that could be construed as a potential conflict of interest.
